# Annual acknowledgement of manuscript reviewers

**DOI:** 10.1186/1471-2229-13-17

**Published:** 2013-02-19

**Authors:** Simon Harold

**Affiliations:** 1 BioMed Central, Floor 6, 236 Grey's Inn Road, London WC1X 8HB, UK

## Abstract

**Contributing reviewers:**

The editors of *BMC Plant Biology* would like to thank all of our reviewers who have contributed to the journal in Volume 12 (2012).

## 

Ibrokhim Abdurakhmonov

Uzbekistan

Wafa Achouak

France

Parvaiz Ahmad

India

Oussama Ahrazem

Spain

Tariq Akhtar

USA

Eduard Akhunov

USA

Merete Albrechtsen

Denmark

Francisco Alfocea

Spain

Nadim Alkharouf

USA

Yagut Allahverdiyeva

Finland

Randy Allen

USA

Maneesha Aluru

USA

Sara Amancio

Portugal

Toyoaki Anai

Japan

Karel J Angelis

Czech Republic

Heidi Appel

USA

Felipe Aquea

USA

Gen-ichiro Arimura

Japan

Patrick Armengaud

France

Ricardo Aroca

Spain

Rajeev Arora

USA

Jose T. Ascencio-Ibanez

USA

Maria Asins

Spain

Michael Axtell

USA

Brian Ayre

USA

Driouich Azeddine

France

Eleni Bachlava

USA

Charles Bacon

USA

Spela Baebler

Slovenia

Yuling Bai

Netherlands

Donovan Bailey

USA

Sureshkumar Balasubramanian

Australia

Raffaella Balestrini

Italy

Peter Balint-Kurti

USA

Frantisek Baluska

Germany

Antoni Banas

Poland

Zsofia Banfalvi

Hungary

Simon Barak

Israel

Abdelali Barakat

USA

Younas Barozai

Pakistan

Philippe Barre

France

Ramamurthy Baskar

India

Roberto Bassi

Italy

Jacqueline Batley

Australia

Luigi Bavaresco

Italy

Manuel Becana

Spain

Jorg Becker

Portugal

Piotr Bednarek

Poland

Eric Beers

USA

Mohammed Bendahmane

France

Philip Benfey

USA

Moussa Benhamed

France

Alan Bennett

USA

Malcolm John Bennett

UK

Jeffrey Bennetzen

USA

Alison Berry

USA

David Bertioli

Brazil

Magdalena Bezanilla

USA

Craita E Bita

Netherlands

Petra Bleeker

Netherlands

Burt Bluhm

USA

Joerg Bohlmann

Canada

Melvin Bolton

USA

Gustavo Bonaventure

Germany

Donna Bond

UK

Claudio Bonghi

Italy

Guusje Bonnema

Netherlands

Wendy Boss

USA

Alessandro Botton

Italy

Andrew Bowling

USA

Lewis Bowman

USA

Peter Bozhkov

Sweden

Federica Brandizzi

USA

Anja Branscheid

Denmark

Alan Brash

USA

James Breen

Switzerland

Adina Breiman

Israel

Oliver Brendel

France

Adrian Brennan

Spain

Karen Browning

USA

Teerapong Buaboocha

Thailand

Etienne Bucher

Switzerland

Marcel Bucher

Germany

Lorenz Bülow

Germany

Daniel Bush

USA

Victor Busov

USA

Melinka Butenko

Norway

Xinjiang Cai

USA

Pat Calie

USA

Ann Callahan

USA

Judy Callis

USA

Hector Candela

Spain

Thierry Candresse

France

Daniela Capiati

Argentina

Emilio A. Carbonell Guevara

Spain

Anders Carlsson

Sweden

Bernard Carroll

Australia

Carla Caruso

Italy

Josep Casacuberta

Spain

Almudena Castillo

Spain

Mariana Castro

Brazil

Luigi Cattivelli

Italy

Eva Cellarova

Slovakia

Claudio Cerboncini

Germany

Stefano Cesco

Italy

Lee Chae

USA

David Chagne

New Zealand

Joe Chappell

USA

Alain Charcosset

France

Rajinder Chauhan

India

Aakash Chawade

Sweden

Feng Chen

USA

Xianming Chen

USA

Charles Chen

USA

Rujin Chen

USA

Zhixiang Chen

USA

Christian Chervin

France

Tzen-Yuh Chiang

Taiwan

Tzyy-Jen Chiou

Taiwan

Hye Sun Cho

South Korea

Surinder Chopra

USA

Don Cipollini

USA

Nicole Clay

USA

Luca Comai

USA

Peter Constabel

Canada

Fabrizio Costa

Italy

Rocio Cruz-Ortega

Mexico

Yuehua Cui

USA

Tomasz Czechowski

UK

Henryk Czosnek

Israel

Nunzio D’Agostino

Italy

Tim Daniell

UK

Margaret Daub

USA

Barend de Graaf

UK

Wanessa De Lima

Switzerland

Giulia De Lorenzo

Italy

Emanuele De Paoli

Italy

Daniel G. Debouck

Colombia

Joanna Deckert

Poland

Veronique Decroocq

France

John Paul Delano-Frier

Mexico

Philippe Delavault

France

Alia Dellagi

France

Serge Delrot

France

Taku Demura

Japan

Katherine Denby

UK

France Denoeud

France

Alessandra Devoto

UK

Michael K Deyholos

Canada

Rahul Dhawan

USA

Amit Dhingra

USA

Gabriele Di Gaspero

Italy

Adele di Matteo

Italy

Maria L. Diaz Chavez

Canada

Anne Dievart

France

Gianfranco Diretto

Italy

Robert Doczi

Hungary

James Dombrowski

USA

David Domozych

USA

Aiwu Dong

China

John Doonan

UK

Berta Dopico

Spain

Noelle Dorion

France

Carl Douglas

Canada

Xavier Draye

Belgium

Jean-Jaques Drevon

France

Maria F Drincovich

Argentina

Zhiyou Du

China

Qiaohong Duan

USA

Karl Duffy

South Africa

Paul Dupree

UK

Atsushi Ebihara

Japan

Johan Edqvist

Sweden

David Edwards

Australia

Marcos Egea-Cortines

Spain

Luis E. Eguiarte

Mexico

Malin Elfstrand

Sweden

Paula Elomaa

Finland

Khatere Emadzade

Austria

Francois Eudes

Canada

Daniele Evers

Luxembourg

Hiroshi Ezura

Japan

Vasiliki Falara

USA

Jinggui Fang

China

Melanie Febrer

UK

Urs Feller

Switzerland

Trevor Fenning

UK

Morag Ferguson

Kenya

Paulo Ferreira

Brazil

John Finer

USA

Victor Flors

Spain

Robert Fluhr

Israel

Rhonda Foley

Australia

Fabio Fornara

Italy

Perez Francisco

Chile

Philipp Franken

Germany

Elisabetta Frascaroli

Italy

Ceridwen Fraser

Belgium

Swetlana Friedel

Germany

Julia Frugoli

USA

Xiangdong Fu

China

Jinmin Fu

China

Yong-Fu Fu

China

Yong-Bi Fu

Canada

Jianming Fu

USA

Atsushi Fukushima

Japan

Lynette Fulton

Australia

Jose Gadea

Spain

Oliver Gailing

USA

Patrick Gallois

UK

Qingming GAO

USA

Caiji Gao

Hong Kong

Dongying Gao

USA

Andrea Garavito

Colombia

Susan Gardiner

New Zealand

Kim Garland Campbell

USA

Charles Gasser

USA

Walter Gassmann

USA

Helene Gautier

France

Sonia Gazzarrini

Canada

Bernard Genty

France

Marcelo German

USA

Hernâni Gerós

Portugal

Navdeep Gill

Canada

Matthew Gilliham

Australia

Stewart Gillmor

Mexico

Carmina Gisbert

Spain

Giovanni Giuliano

Italy

Stefan Gleissberg

USA

Fred Gmitter

USA

Zhizhong Gong

China

Clara Isabel Gonzalez Verdejo

Spain

Julie Graham

UK

Lydia Gramzow

Germany

Silvana Grandillo

Italy

Barbara Gravendeel

Netherlands

John Gray

USA

Peter Gresshoff

Australia

Jerome Grimplet

Spain

Paul E. Grini

Norway

Delphine Grivet

Spain

Briana Gross

USA

Rita Gross-Hardt

Germany

Michael Groszmann

Australia

Kristina Gruden

Slovenia

Rebecca Grumet

USA

Juan Guiamet

Argentina

Yi Guo

China

Kapuganti J Gupta

UK

Jeffery Gustin

USA

Heidi Halbwirth

Austria

Nigel Halford

UK

Bjoern Hamberger

Denmark

Patrice Hamel

USA

John Hammond

Australia

Yuepeng Han

China

Kousuke Hanada

Japan

Robert Hancock

UK

Roger Hangarter

USA

David Hannapel

USA

Abdelali Hannoufa

Canada

John Harada

USA

Neil Harris

Canada

Wendy Harwood

UK

Bettina Hause

Germany

Mike Haydon

UK

Guangyuan He

China

Sheng Yang He

USA

Xin-Jian He

China

Junxian He

Hong Kong

Guohao He

USA

Kim Hebelstrup

Denmark

Alain Hehn

France

Ingo Heilmann

Germany

Jochen Heinrichs

Germany

Jan Hejatko

Czech Republic

Chris Helliwell

Australia

Lars Hennig

Sweden

Rossana Henriques

USA

Robert Henry

Australia

Alexander Heyl

Germany

Andreas Hiltbrunner

Germany

Dirk Hincha

Germany

Shailaja Hittalmani

India

Owen Hoekenga

USA

A. Scott Holaday

USA

David Honys

Czech Republic

Stefan Hoth

Germany

Andreas Houben

Germany

Dianella Howarth

USA

Jinguo Hu

USA

Xiangyang Hu

China

Jianping Hu

USA

Zhihua Hua

USA

Rongfeng Huang

China

Jinling Huang

USA

Ralph Hueckelhoven

Germany

Scot Hulbert

USA

Ildoo Hwang

South Korea

Inhwan Hwang

South Korea

Yoko Iijima

Japan

Richard Immink

Netherlands

Rob Ingle

South Africa

Yoshiaki Inukai

Japan

Muhammad Iqbal

USA

Brian Irish

United States Minor Outlying Islands

Judith Irwin

UK

Tal Isaacson

Israel

Nurul Islam-Faridi

USA

Aiko Iwata

USA

Thomas Jack

USA

Guru Jagadeeswaran

USA

Andrew James

Mexico

E Jamet

France

Sravan Jami

Canada

Tibor Janda

Hungary

Cheol Seong Jang

South Korea

Bart Janssen

New Zealand

Artur Jarmolowski

Poland

Paul Jarvis

UK

Michal Jasinski

Poland

Sylvain Jeandroz

France

Dong-Hoon Jeong

USA

Bhavanath Jha

India

Wensuo Jia

China

Xiaoyun Jia

China

Yuanqing Jiag

China

Ning Jiang

USA

Yuling Jiao

China

Dominique Job

France

Frank Johannes

Netherlands

Eric Johnson

USA

Huw Jones

UK

Alan Jones

USA

Maarten Jongsma

Netherlands

Arun Joshi

India

Christian Jung

Germany

Shawn Kaeppler

USA

Akito Kaga

Japan

Ralf Kaldenhoff

Germany

Hunseung Kang

South Korea

Merijn Kant

Netherlands

Michael Kantar

USA

Ching-Huei Kao

Taiwan

Sanjay Kapoor

India

Rupesh Kariyat

USA

Anna Kasprowicz

Poland

Maki Katsuhara

Japan

Ravneet Kaur

Canada

Shigeyuki Kawano

Japan

Kemal Kazan

Australia

Beat Keller

Switzerland

Stefan Kempa

Germany

Sekine Ken-Taro

Japan

Muhammad Awais Khan

USA

Shoshi Kikuchi

Japan

Jae-Yean Kim

South Korea

Soo Young Kim

South Korea

Graham King

Australia

Tetsu Kinoshita

Japan

Helmut Kirchhoff

USA

Vinod KK

India

Volker Knoop

Germany

Takao Koeduka

Japan

Michael Kolomiets

USA

Anna Koltunow

Australia

Setsuko Komatsu

Japan

Stanislav Kopriva

UK

Simeon Kotchoni

USA

Elena Kramer

USA

Kirsten Krause

Norway

Beth Krizek

USA

Hanhui Kuang

China

Prakash Kumar

Singapore

Helge Küster

Germany

John Labavitch

USA

Zhibing Lai

USA

Shailesh Lal

USA

Daryl Lam

USA

Laurent Laplaze

France

Colette Larrre

France

Debbie Laudencia-Chingcuanco

USA

David Lee

UK

Wing-Sham Lee

UK

Richard Leegood

UK

Michel Legrand

France

Fabiola Leon

Mexico

Anne-Sophie Leprince

France

Amnon Lers

Israel

Krzysztof Lesniewicz

Poland

Gerhard Leubner

UK

Efraim Lewinsohn

Israel

Mathew G Lewsey

USA

Jiarui Li

American Samoa

Jiarui Li

USA

Xiu-Qing Li

Canada

Hsou-min Li

Taiwan

Ling Li

USA

Xinguo Li

Australia

Haiying Liang

USA

Marc Libault

USA

Wilco Ligterink

Netherlands

Diego Lijavetzky

Argentina

Hong-Hui Lin

China

Huub Linthorst

Netherlands

Vincenzo lionetti

Italy

Aizhong Liu

China

Liwang Liu

China

Jin-Yuan Liu

China

Bao Liu

China

Kede Liu

China

Chang-Jun Liu

USA

Clive Lo

Hong Kong

Gary Loake

UK

Franca Locatelli

Italy

Wayne Loescher

USA

Jason Londo

USA

Camilo Lopez

Colombia

Ana-Flor Lopez-Millan

Spain

Tiago Lourenço

Portugal

John Love

UK

Gang Lu

China

Yongxian Lu

USA

Thomas Lübberstedt

USA

Lewis Lukens

Canada

Hong Luo

USA

Henrik Lütken

Denmark

Doan Trung Luu

France

Martin A. Lysak

Czech Republic

Chuanxi Ma

China

Wujun Ma

Australia

Lu Ma

Germany

Fengwang Ma

China

John MacKay

Canada

Elena Maestri

Italy

Massimo Maffei

Italy

Izabela Makalowska

Poland

Robert Malinowski

UK

Mickael Malnoy

Italy

Bohumil Mandak

Czech Republic

Shoji Mano

Japan

Johanna Mansfeld

Germany

Nitin Mantri

Australia

Shrikant Mantri

India

Rogerio Margis

Brazil

Marcia Margis

Brazil

Ferenc Marincs

Hungary

Annie Marion-Poll

France

M. David Marks

USA

Daniela Marone

Italy

Antonio j Marquez

Spain

Stefan Martens

Italy

Ruth Martin

USA

Susana Martin Ortigosa

USA

Jan Martinec

Czech Republic

Jose M. Martinez-Rivas

Spain

Liliana Marum

Portugal

Angelo Matilla

Spain

Kazuhiko Matsuda

Japan

Satoru Matsumoto

Japan

Sachihiro Matsunaga

Japan

Yoshihiro Matsuoka

Japan

Benjamin Matthews

USA

José Tomás Matus

Spain

Radoslava Matusova

Slovakia

Felix Mauch

Switzerland

Brigitte Mauch-Mani

Switzerland

Martin Robert McAinsh

UK

Phillip McClean

USA

Stuart McDaniel

USA

Mitch McGrath

USA

Leah McHale

USA

Hanwei Mei

China

Harold Meijer

Netherlands

David Meinke

USA

Ewa Mellerowicz

Sweden

Jinling Meng

China

Livia Merendino

France

Irute Meskiene

Austria

Rainer Messmer

Switzerland

Sofiane Mezmouk

USA

Dominique Michaud

Canada

Célia Miguel

Portugal

Andrew Millar

UK

Norihiko Misawa

Japan

Juliane Mittasch

Germany

Hiroshi Miyake

Japan

Todd Mockler

USA

Wolfgang Moeder

Canada

Lorena Moeller

USA

Christian Möllers

Germany

Jean-Benoit Morel

France

Marcio de Carvalho Moretzsohn

Brazil

Masaki Mori

Japan

Thomas Morris

USA

Agnieszka Mostowska

Poland

Mario motto

Italy

Zhonglin Mou

USA

Said Mouzeyar

France

Gary Muehlbauer

USA

Lukas Mueller

USA

Bernd Mueller-Roeber

Germany

Carsten Muessig

Germany

Robert Mullen

Canada

Yoshiyuki Murata

Japan

Irene Murgia

Italy

Alex Murphy

UK

Angus Murphy

USA

Jorge Muschietti

Argentina

Michael Muszynski

USA

Marco Muzi-Falconi

Italy

Sean Myles

USA

Joshua Mylne

Australia

Eiji Nambara

Canada

Guo-Ling Nan

USA

Judith Nardmann

Germany

Andreas Nebenführ

USA

Jennifer Nemhauser

USA

Jean-Marc Neuhaus

Switzerland

Ed Newbigin

Australia

Zhongfu Ni

China

Paul Nicholson

UK

Scott Nicholson

USA

Totte Niittylä

Sweden

Naoko Nishizawa

Japan

Shingtok Niu

China

Michael Nodine

Austria

Helen North

France

Evandro Novaes

Brazil

Hisayoshi Nozaki

Japan

Sergio J Ochatt

France

Erich-Christian Oerke

Germany

Man-Ho Oh

USA

Halley Oliveira

Brazil

Kenneth Olsen

USA

Carmel O’Neill

UK

David Oppenheimer

USA

Ariel Orellana

Chile

Colin Orians

USA

Jamie O’Rourke

USA

Rodomiro Ortiz

Sweden

Takashi Osono

Japan

Chris Owens

USA

Peggy Ozias-Akins

USA

Jonathan Page

Canada

Ravishankar Palanivelu

USA

Sona Pandey

USA

Tiziana Pandolfini

Italy

Ralph Panstruga

Germany

Ashwani Pareek

India

Basavaprabhu Patil

USA

John Patrick

Australia

Robert Paull

USA

Markus Pauly

USA

Katharina Pawlowski

Sweden

Kerry Pedley

USA

Steven Penfield

UK

Jianling Peng

USA

Michele Perazzolli

Italy

Carla Perrotta

Italy

Marko Petek

Slovenia

Gitte Petersen

Denmark

Mario Pezzotti

Italy

Andy Phillips

UK

Eran Pichersky

USA

Hans-Peter Piepho

Germany

Ronald Pierik

Netherlands

Corné Pieterse

Netherlands

Paula Pijut

USA

Peter Pimpl

Germany

Fabio Pinheiro

Brazil

Elisabeth Planchet

France

Dorina Podar

Romania

Florencio Podestá

Argentina

Benoit Poinssot

France

Annalisa Polverari

Italy

Federico Pomar

Spain

Inés Ponce de León

Uruguay

Brigitte Poppenberger

Germany

Zoe Popper

Ireland

Ezio Portis

Italy

Ann Powell

USA

Tony Pridmore

UK

Dirk Prüfer

Germany

Holger Puchta

Germany

Michael Puckette

USA

Sergi Puig

Spain

Naveen Puppala

USA

Jo Putterill

New Zealand

Cheng Qin

China

Wenping Qiu

USA

Lijuan Qiu

China

Antoni Rafalski

USA

Ramesh Raina

USA

K Rajasekaran

USA

C Raman

USA

Nirala Ramchiary

South Korea

Angelo Ramina

Italy

Luke Ramsay

UK

Punna Ramu

India

Harpinder Randhawa

Canada

Amanda Rasmussen

Belgium

Deborah Rathbone

UK

Michael Reagon

USA

Fabrice Rébeillé

France

John Reese

USA

Rob Reid

Australia

Shuxin Ren

USA

Susanne Renner

Germany

Stefan Rensing

Germany

Philippe Reymond

Switzerland

René Richter

Germany

Dae-Kyun Ro

Canada

Jeremy Roberts

UK

Marion Roder

Germany

Hilary Rogers

UK

Charles Romieu

France

Ann Christin Rönnberg-Wästljung

Sweden

Fred Rook

Denmark

Ray J. Rose

Australia

Josep A. Rossello

Spain

Christophe Rothan

France

John Runions

UK

Paul Rushton

USA

Scott Russell

USA

Choong-Min Ryu

USA

Yokoyama Ryusuke

Japan

Sabrina sabatini

Italy

Einat Sadot

Israel

Francesco Salamini

Italy

Jozef Samaj

Czech Republic

Ajay Sandhu

USA

Geir Kjetil Sandve

Norway

Mirella Sari Gorla

Italy

Neera Sarin

India

Ananda K Sarkar

India

Yutaka Sato

Japan

Christof Sautter

Switzerland

Sigal Savaldi-Goldstein

Israel

Arnould Savoure

France

Shinichiro Sawa

Korea, North

Olga Sayanova

UK

Enrico Scarpella

Canada

Wilhelm Schaefer

Germany

Robert Schaffer

New Zealand

Ulrich Schaffrath

Germany

Roland Schafleitner

Peru

G. Eric Schaller

USA

Judith Scharte

Germany

Henrik Scheller

USA

Günther Scherer

Germany

John Schiefelbein

USA

Peter Schloegelhofer

Austria

Eric Schmelz

USA

Markus Schmid

Germany

Klaus Schmidt

Germany

Hanna Schneeweiss

Austria

James Schoelz

USA

Stefan Scholten

Germany

Uwe Scholz

Germany

Mark Aurel Schöttler

Germany

Robert Schuurink

Netherlands

Kathy Schwinn

New Zealand

Steven Scofield

USA

Paul Scott

USA

Iain Searle

Australia

Luca Sebastiani

Italy

Juan Segura

Spain

Rajandeep Sekhon

USA

Gopalan Selvaraj

Canada

Mitsunori Seo

Japan

Kjell Sergeant

Luxembourg

Guido Sessa

Israel

Andrew Severin

USA

Dilip Shah

USA

Jyoti Shah

USA

Qingli shang

China

Chun Shi

Canada

Toshiharu Shikanai

Japan

Kentaro Shimizu

Switzerland

Jae-Ho Shin

South Korea

Dorothy Shippen

USA

Jay Shockey

USA

Qing yan Shu

China

Rolf Siegwolf

Switzerland

Rodrigo Singer

Brazil

Sneh Singla-Pareek

India

Rungaroon Waditee Sirisattha

Thailand

Tanja Slotte

Sweden

David Smyth

Australia

Wayne Snedden

Canada

David Somers

USA

Xiang Song

USA

Simon Southerton

Australia

Imogen Sparkes

UK

Cornelia Spetea

Sweden

David Spooner

USA

Otmar Spring

Germany

Nathan Springer

USA

Patricia Springer

USA

Gary Stacey

USA

A. Michele Stanca

Italy

Ann Stapleton

USA

Andre Steinmetz

Luxembourg

C Neal Stewart

USA

Åsa Strand

Sweden

Steven Strauss

USA

Nathaniel Street

Sweden

Martina Strömvik

Canada

Thomas Stützel

Germany

Chun-lin Su

Taiwan

Zhen Su

China

Maria C Suarez Rodriguez

USA

Senthil Subramanian

USA

Genlou Sun

Canada

Sibum Sung

USA

Steve Swain

Australia

Robert Swanson

USA

Ewa Swiezewska

Poland

László Szabados

Hungary

Teruaki Taji

Japan

Teruhiro Takabe

Japan

Yohsuke Takahashi

Japan

Akira Takahashi

Japan

Adam Takos

Denmark

Shigeo Takumi

Japan

Earl Taliercio

USA

Manuel Talon

Spain

Xiaoping Tan

USA

Guiliang Tang

USA

Karen tanino

Canada

Celine tasset

Australia

Akira Tateishi

Japan

Frans Tax

USA

Jane Taylor

UK

Francisco Tenllado

Spain

Evelyne Téoulé

France

Hernan Terenzi

Brazil

Valeria Terzi

Italy

Pilar S. Testillano

Spain

Guenter Theissen

Germany

Marie Thibaud

France

Patrice This

France

Leeann Thornton

USA

Li Tian

USA

Dacheng Tian

China

Bradley Till

Austria

Gail Timmerman-Vaughan

New Zealand

Sivananda Tirumalaraju

USA

Henrik Tjellström

USA

Ramon Torres-Ruiz

Germany

Timothy Tranbarger

France

Ben Trevaskis

Australia

Maria Jose Truco

USA

Athanasios Tsaftaris

Greece

Ching-Hsiu Tsai

Taiwan

Hirokazu Tsukaya

Japan

Colin Turnbull

UK

Ludmila Tyler

USA

Luis Valledor

Austria

Renier van der Hoorn

Germany

Henk Van Dijk

France

Roeland van Ham

Netherlands

Mieke Van Lijsebettens

Belgium

Gerben van Ooijen

UK

Martijn van Zanten

Netherlands

Imre Vass

Hungary

Leonardo Velasco

Sri Lanka

Gruber Veronique

France

Carlos M Vicient

Spain

Sabina Vidal

Uruguay

Maria Lucia C. Vieira

Brazil

Eva Vincze

Denmark

Alessandro Vitale

Italy

Diter von Wettstein

USA

Juan Vorster

South Africa

Ruth Wagner

Netherlands

Michael H. Walter

Germany

Jianmin Wan

China

Kan Wang

USA

Tai Wang

China

Xiu-Jie Wang

China

Ming-Bo Wang

Australia

Guoying Wang

China

Yaosheng Wang

Denmark

Jia-Wei Wang

China

Jiankang Wang

China

Yuejin Wang

China

Preeya Wangsomnuk

Thailand

Scott Warnke

USA

Katherine Warpeha

USA

Allison Weber

USA

Shu Wei

Canada

Dolf Weijers

Netherlands

Clifford Weil

USA

Andreanna Welch

USA

Li Wen-Xue

China

Randall Weselake

Canada

James Westwood

USA

Derek White

New Zealand

Steven Whitham

USA

Christie Williams

USA

Joseph Williams

USA

Roger Wise

USA

Bogdan Wolko

Poland

Thomas Wolpert

USA

Ernst Woltering

Netherlands

Rafal Woycicki

Canada

Yongsheng Wu

China

Ping Wu

China

Jian Wu

China

Martin Wubben

USA

Xinli Xia

China

Xianchun Xia

China

Zhengjun Xia

China

Gui-Xian Xia

China

Yu Xiang

Canada

Shi Xiao

China

Zhixin Xie

USA

Zhanguo Xin

USA

Dong Xu

USA

Guohua Xu

China

Ram Kishor Yadav

India

Yueming Yan

China

Shuichi Yanagisawa

Japan

Yinong Yang

USA

Chang-Hsien Yang

Taiwan

Tae-Jin Yang

South Korea

Hongquan Yang

China

Hai-ling Yang

China

Zhi Min Yang

China

Kuo-Chen Yeh

Taiwan

Charles Yocum

USA

Riichiro Yoshida

Japan

Bin Yu

USA

Xiaolin Yu

China

Joshua Yuan

USA

Olga Zabotina

USA

Mirko Zaffagnini

Italy

Viktor Zarsky

Czech Republic

Fei Zhang

China

Yan Zhang

China

Shuqun Zhang

USA

Zhanyuan Zhang

USA

Xianlong Zhang

China

Xian Sheng Zhang

China

Jin-Song Zhang

China

Baohong Zhang

USA

Li-Wu Zhang

China

Peng Zhang

China

Dazhong Zhao

USA

Zhong Zhao

China

Ying Zhu

China

Shifeng Zhu

USA

Longfu Zhu

China

Yanming Zhu

China

Shunyi Zhu

China

Heiko Ziebell

Germany

Laura Zsigmond

Hungary

Andrea Zuccolo

Italy

Janusz Zwiazek

Canada

Eva Zyprian

Germany

